# Small Extracellular Vesicles Orchestrate Cisplatin‐Induced Ototoxicity: Potential Biomarker and Targets Discovery

**DOI:** 10.1002/advs.202502627

**Published:** 2025-05-24

**Authors:** Jingru Ai, Shasha Zhang, Mingchen Dai, Pei Jiang, Jingyuan Huang, Hairong Xiao, Yanqin Lin, Xujun Tang, Wei Tong, Jun He, Qiuyue Mao, Yintao Wang, Zixuan Ye, Tian Wang, Renjie Chai

**Affiliations:** ^1^ State Key Laboratory of Digital Medical Engineering Department of Otolaryngology Head and Neck Surgery Zhongda Hospital School of Life Sciences and Technology Advanced Institute for Life and Health Jiangsu Province High‐Tech Key Laboratory for Bio‐Medical Research Southeast University Nanjing 210096 China; ^2^ Southeast University Shenzhen Research Institute Shenzhen 518063 China; ^3^ Department of Otolaryngology – Head and Neck Surgery The Second Xiangya Hospital Central South University Changsha Hunan 410011 China; ^4^ Department of Otolaryngology – Head and Neck Surgery Stanford University School of Medicine Stanford CA 94305 USA; ^5^ Department of Otolaryngology Head and Neck Surgery Sichuan Provincial People's Hospital School of Medicine The University of Electronic Science and Technology of China Chengdu 610072 China; ^6^ Co‐Innovation Center of Neuroregeneration Nantong University Nantong 226001 China; ^7^ Institute for Stem Cells and Regeneration Chinese Academy of Science Beijing 100081 China

**Keywords:** biomarker, cisplatin‐induced ototoxicity, CLTC, miRNAs, small extracellular vesicles

## Abstract

Cisplatin‐induced ototoxicity remains a clinical dilemma with limited mechanistic understanding and no food and drug administration (FDA)‐approved therapies. Despite emerging roles of small extracellular vesicles (sEV) in drug ototoxicity, their molecular cargo profiles and causal roles to cisplatin‐induced ototoxicity are unexplored. This study systematically investigates sEV derived from cochlear explants treated with cisplatin (Cis‐sEV) and controls (Ctrl‐sEV) using multi‐omics profiling. Through small RNA sequencing, 83 differentially expressed microRNAs (miRNAs) are identified in Cis‐sEV compared to Ctrl‐sEV. Notably, mmu‐miR‐34a‐5p, mmu‐miR‐140‐5p, mmu‐miR‐15b‐5p, mmu‐miR‐25‐3p, and mmu‐miR‐339‐5p are significantly upregulation in Cis‐sEVs. Predicted target pathways of these differentially expressed miRNAs are enriched in apoptosis, inflammation, and cellular damage, indicating their potential involvement in cisplatin‐induced cochlear damage. LC‐MS/MS analysis reveals 90 upregulated and 150 downregulated proteins in Cis‐sEV, with many involved in damage response. Specifically, CLTC, CCT2, ANXA6, and HSPA8 are uniquely upregulated proteins in Cis‐sEV, and CLTC and ANXA6 are exclusively co‐localized in hair cells (HCs) post‐cisplatin exposure, suggesting that Cis‐sEV originate primarily from damaged HCs. Moreover, CLTC in sEV may serve as a potential biomarker for cisplatin‐induced ototoxicity as verified in both in vitro and in vivo models. This study provides novel insights into the molecular mechanisms of cisplatin‐induced ototoxicity and identifies potential biomarker and therapeutic targets.

## Graphical abstract

Cisplatin causes reactive oxygen species accumulation, leading to apoptosis and inflammation in cochlear hair cells. Small extracellular vesicles primarily derived from the damaged hair cells likely contribute to cisplatin‐induced ototoxicity, carrying a variety of microRNAs and proteins. These differentially expressed components found in these cisplatin‐derived small extracellular vesicles may serve as biomarkers and potential targets for mitigating cochlear damage.

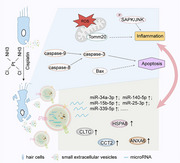

## 1 Introduction

Hearing is crucial for social activities but is particularly vulnerable to factors like genetics, ototoxic drugs, noise exposure, and aging, leading to irreversible sensorineural hearing loss (SNHL).^[^ ^1^ ^]^ Cisplatin, approved by the food and drug administration (FDA) in 1978, is a potent chemotherapeutic drug for malignant solid tumors but often causes severe adverse effects, including bilateral, progressive, and irreversible ototoxicity in 40% to 60% of patients.^[^ ^2^ ^]^

Cisplatin‐induced ototoxicity predominantly results in injury to hair cells (HCs), stria vascularis (SVs), and spiral ganglion neurons (SGNs) within the inner ear. Moreover, the basal turn of the cochlea, which is particularly sensitive to high‐frequency signals, is the initial damage site of cisplatin ototoxicity and suffers the most severe damage.^[^ ^3^ ^]^ High‐dose accumulation of cisplatin can also compromise auditory cells' apical and middle regions. Previous investigations have shown that apoptosis and inflammation elicited by reactive oxygen species (ROS), necrosis, ferroptosis, and the accumulation of lipid peroxides are involved in the progression of cisplatin ototoxicity.^[^ ^4^ ^]^ Nevertheless, the cellular interactions and molecular mechanisms underlying cisplatin‐induced ototoxicity have not yet been completely clarified and still need to be studied.

Small extracellular vesicles (sEV) are lipid bilayer vesicles (<200 nm in diameter) secreted by almost all cell types, and they carry multifarious bioactive molecules that reflect the characteristics of their parent cells.^[^ ^5^ ^]^ sEV‐mediated intercellular communication is a highly conserved process involving the transfer of functional molecules to recipient cells that are nearby or distant (via biofluids and circulation).^[^ ^5^ ^]^ Increasing evidence indicates that sEVs play a significant role in physiological and pathological processes, including the development and progression of cancer, neurodegenerative disease progression, immune regulation, cell survival, and apoptosis.^[^ ^6^ ^]^ In addition, sEV exhibit heterogeneity, primarily characterized by variations in their cargoes of proteins and microRNAs (miRNAs).^[^ ^7^ ^]^ Under various stress conditions and pathological states, this diversity becomes particularly pronounced, prompting sEV to potentially serve as disease biomarkers for monitoring specific disease development or assisting in diagnosis.^[^ ^8^ ^]^

sEVs are critical mediators of intercellular crosstalk in the inner ear,^[^ ^9^ ^]^ yet their molecular specificity in cochlear pathologies remains enigmatic. While a recent study revealed sEV‐mediated protection of vestibular HCs against aminoglycoside toxicity,^[^ ^10^ ^]^ the cochlea – an anatomically complex microenvironment – exhibits fundamentally distinct sEV dynamics under cisplatin‐induced ototoxicity. Crucially, the cargo composition (miRNAs/proteins) of sEV secreted in response to cisplatin‐induced ototoxicity remains unclear,^[^ ^9a^ ^]^ and whether these cisplatin‐modified sEV actively drive sensory cell death and reflecting damage, which are the main focuses of this study. A deeper understanding of the role of sEV in cisplatin‐induced ototoxicity is imperative to developing effective strategies to prevent or mitigate cisplatin's ototoxic effects and therapy optimization for chemotherapy patients.

This study analyzed the miRNAs and proteins from organotypic cochlea‐secreted sEV in cisplatin‐induced ototoxicity and their potential roles in cisplatin‐induced damage. Our findings indicate that the differentially expressed miRNAs in the Cis‐sEV group probably mediate several important cellular and molecular regulation pathways – such as apoptosis, inflammation, and cell survival – which provides a novel perspective for elucidating the downstream effects that contribute to the cochlea's unique vulnerability to cisplatin‐induced injury. Furthermore, several sEV miRNAs are being verified and might be potential therapeutic targets for mitigating cisplatin ototoxicity. Additionally, through LC‐MS/MS analysis and validation, we identified four proteins – clathrin heavy chain 1 (CLTC), T‐complex protein 1 subunit beta (CCT2), annexin A6 (ANXA6), and heat shock cognate 71 kDa protein (HSPA8) – that exhibited a specifical increase expression in Cis‐sEV in the cochlea explant model. Moreover, the damaged HCs are primarily the parent cells of Cis‐sEV, as indicated by the exclusively co‐localized of CLTC and ANXA6. By using the cisplatin‐induced House Ear Institute‐Organ of Corti 1 (HEI‐OC1) cell model and in vivo mice model, CLTC was also enriched expression in Cis‐sEV and could serve as a potential biomarker for cisplatin‐induced ototoxicity. The detailed mechanisms by which the miRNAs and proteins in Cis‐sEV respond to disease progression require further investigation in future studies.

## 2 Results

### 2.1 Establishment of an Ex Vivo Model of Cisplatin‐Induced Ototoxicity

**Figure 1:**
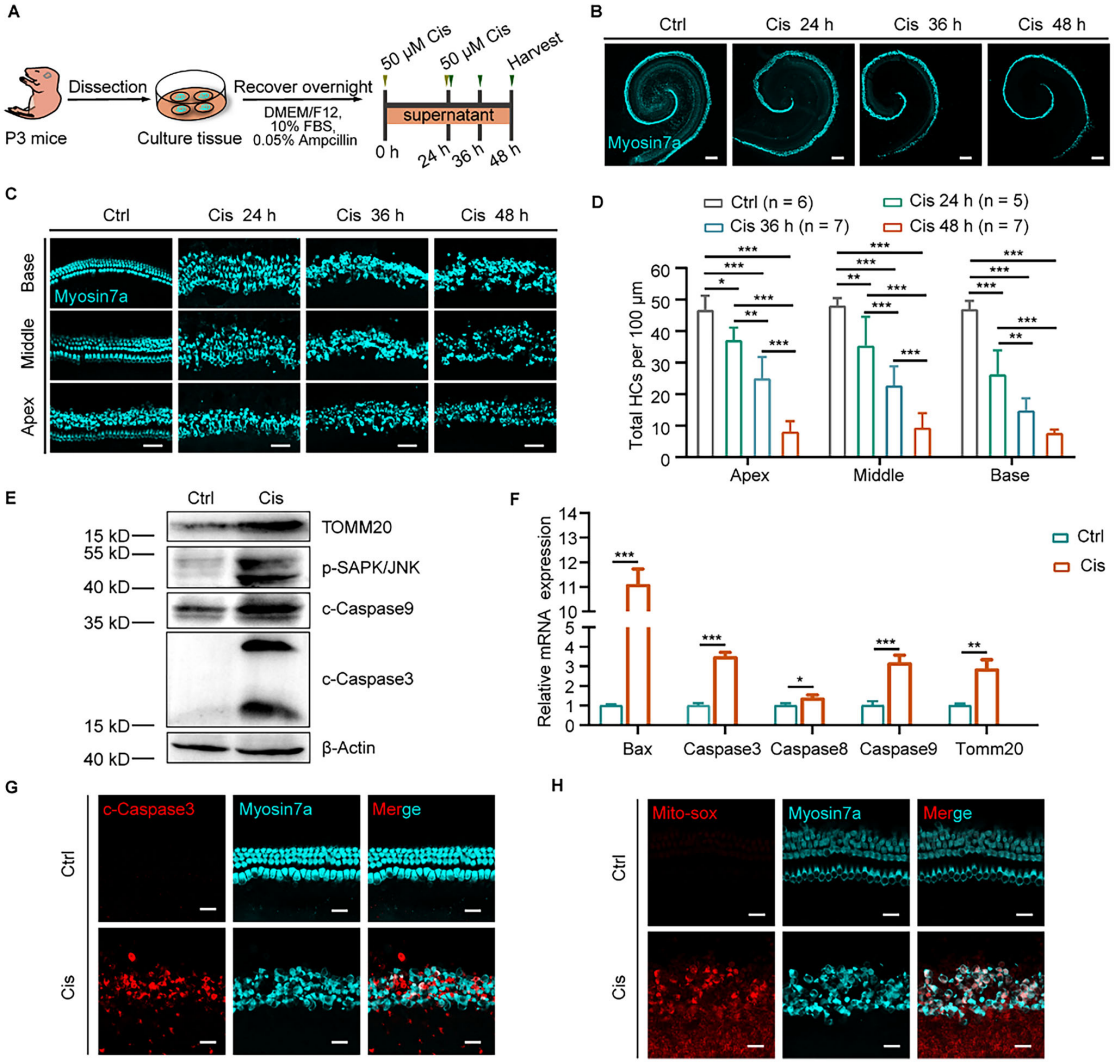
ROS accumulation, apoptosis, and inflammatory responses of auditory HCs in the *ex vivo* cisplatin‐induced ototoxicity model. A) The flow chart of *ex vivo* cochlear explant culture with 50 µM cisplatin. B,C) Representative confocal images of cochlear explants treated with various durations of 50 µM cisplatin (including 24 h, 36 h, and 48 h) are presented, including the complete cochlear explants (B) and local segments of the apical, middle, and basal turns of the cochlear explants (C). The control group of cochlear explants was cultured for 48 h without drug treatment. Myosin7a (cyan) marked HCs. Scale bars are 200 µm (B) and 50 µm (C). D) A quantitative analysis of HCs in each cochlear turn at different cisplatin treatment periods is shown, expressed as the number of HCs per 100 µm of the basilar membrane. Data are shown as mean ± SD, with “*” indicating *p* < 0.05, “**” indicating *p* < 0.01, and “***” indicating *p* < 0.001. “n” represents the number of cochlear explants counted. E) The levels of TOMM20 protein, phosphorylated SAPK/JNK, cleaved‐Caspase3, and cleaved‐Caspase9 in the cochlear explants were all significantly increased after cisplatin‐induced injury at 48 h. β‐Actin served as the reference protein. F) The mRNA levels of *Bax*, *Caspase3*, *Caspase8*, *Caspase9*, and *Tomm20* were significantly increased when the cochlea was exposed to cisplatin for 48 h; n = 3. All results are presented as the mean ± SD, with statistical significance indicated as follows: “*” for *p* < 0.05, “**” for *p* < 0.01, and “***” for *p* < 0.001. G) Immunostaining with cleaved‐Caspase3 (red fluorescence) and anti‐Myosin7a (green fluorescence) showed that HCs in the cisplatin group exhibited a high level of the pro‐apoptosis factor. Scale bar, 20 µm. H) Mito‐Sox fluorescence staining labeled mitochondrial ROS levels in living HCs, showing significant ROS accumulation and oxidative stress in HCs after cisplatin administration. Scale bar, 20 µm.

The cochlear explants from postnatal day 3 (P3) mice were treated with 50 µM cisplatin for 48 h to establish an *ex vivo* model of cisplatin‐induced ototoxicity (**Figure** **1**A). Compared to the control group, the group exposed to 50 µM cisplatin exhibited significant damage to HCs along the cochlear basilar membrane by 24 h, with the severity of damage increasing from the apex to the base, leaving ≈70% of HCs intact (Figure 1B,C,D). As the duration of drug exposure extended to 36 h and 48 h, the resulting damage progressively worsened, leading to a time‐dependent decrease in HC survival rates across all turns of the cochlea (Figure 1B,C,D), which aligns with previous studies.^[^ ^3^, ^4^ ^]^ ≈80% of the HCs in each cochlear turn were lost after 48 h of cisplatin treatment (Figure 1D). Additionally, at 48 h, the protein expression of TOMM20 and p‐SAPK/JNK showed a noticeable increase in the cisplatin group, indicating the accumulation of ROS and an inflammatory response following cisplatin administration (Figure 1E). The accumulation of ROS in the mitochondria of HCs exposed to cisplatin was further confirmed by Mito‐Sox fluorescence staining (Figure 1H). Moreover, the protein level of apoptosis markers, including cleaved‐Caspase9 and cleaved‐Caspase3, was increased in response to cisplatin‐induced injury, as shown by western blotting and immunofluorescence results (Figure 1E,G). This indicates that the damaged HCs underwent apoptosis following cisplatin stress. Furthermore, cisplatin treatment led to a significant increase in the expression of mRNA for pro‐apoptosis genes such as *Bax*, *Caspase3*, *Caspase8*, and *Caspase9*, as well as the ROS‐related gene *Tomm20* (Figure 1F). In summary, we successfully established an *ex vivo* model of cisplatin‐induced ototoxicity, characterized by ROS accumulation, apoptosis, and an inflammatory response.

### 2.2 Isolation and Characterization of Tissue‐Derived sEV from Cisplatin‐Treatment Cochlear Explants

**Figure 2:**
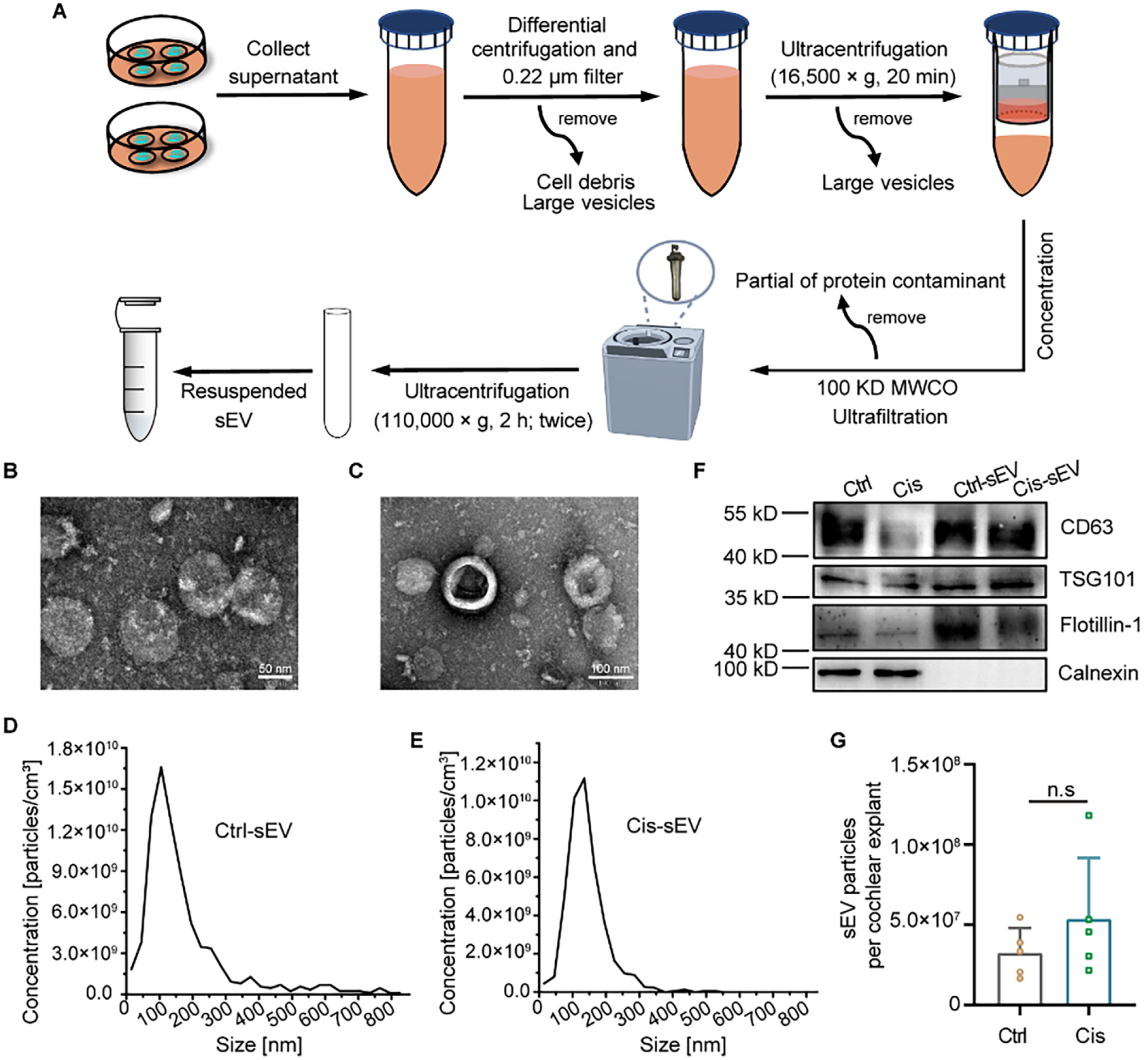
Isolation and characterization of cochlear tissue‐derived sEV form cochlear explants. A) The flow diagram for isolating sEV from the conditioned culture medium of the cochlear explants involves a 48‐h treatment. B,C) TEM analysis of the morphology of Cis‐sEV and Ctrl‐sEV. Scale bars, 50 nm (B) and 100 nm (C). D,E) Particle size distribution of Cis‐sEV and Ctrl‐sEV was determined by NTA. F) Western blotting analysis of isolated sEV and cochlear explants tissue lysate with typical positive markers for sEV, such as CD63, TSG101, and Flotllin‐1, and the negative marker Calnexin, normalized according to BCA protein quantification results. G) Statistical analysis of the number of sEV secreted per cochlear explant in the control group and cisplatin‐treated group. Ctrl group: n = 5 samples; Cis group: n = 5 samples. The “n.s” represent the no signification.

Tissue‐derived sEV are found in the intercellular space of tissues and play significant roles in facilitating intercellular communication within the tissue microenvironment. Therefore, they closely reflect the characteristics of the parental cells and the surrounding tissue environment.^[^ ^11^ ^]^ To investigate changes in sEV secretion patterns associated with the progression of cisplatin‐induced toxicity, we isolated the sEV from the supernatant of the cochlear explant culture medium over a continuous timeframe from 0 to 48 h. This isolation process involved a series of differential centrifugation, ultrafiltration, and ultracentrifugation steps, as illustrated in the flow diagram presented in **Figure** **2**A. The sEVs collected from the cisplatin‐treated group are designated as Cis‐sEV, while those derived from the control group are designated as Ctrl‐sEV. TEM analysis revealed typical oval and cup‐shaped structures in both types of sEVs, with sizes of ≈200 nm (Figure 2B,C). Additionally, the NTA results showed that both Ctrl‐sEV and Cis‐sEV were within the size range of 200 nm (Figure 2D,E). Statistical analysis revealed that the median size of the Ctrl‐sEV group was 149.6 ± 6.461 nm, while the median size for the Cis‐sEV group was 152.0 ± 5.606 nm, showing no significant difference in median size between the two sEV groups (Figure S1, Supporting Information). Additionally, the mean quantity of derived sEV particles per cochlear explant in the control group was measured at 3.27 × 10^7^ ± 1.52 × 10^7^, compared to 5.38 × 10^7^ ± 3.80 × 10^7^ sEV particles per cochlear explant in the cisplatin‐treated group, also demonstrating no statistical difference between the two groups (Figure 2G). Typical protein markers of sEV (CD63, TSG101, and Flotillin‐1) were confirmed by western blotting in both the isolated sEV and tissue lysate, while Calnexin, an sEV‐negative marker, was detected only in the tissue lysate and not in either the Cis‐sEV or Ctrl‐sEV groups (Figure 2F). Taken together, these results demonstrated that sEVs were effectively isolated and purified from the conditioned culture medium of cochlear explants, which could be used for further research.

### 2.3 Differential miRNA Profiles in Cis‐sEV Compared to Ctrl‐sEV

**Figure 3:**
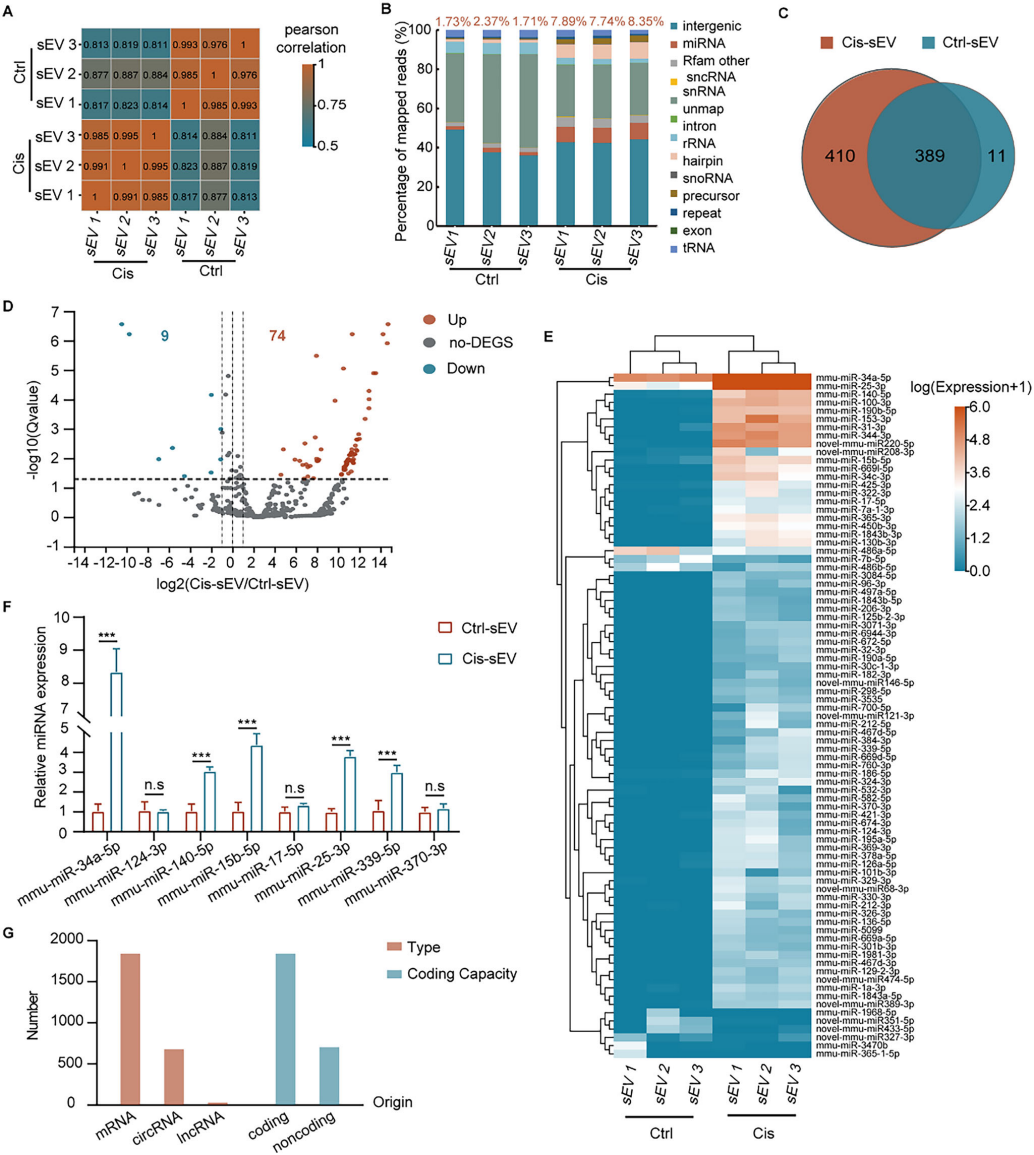
Small RNA‐seq profile and analysis in the Cis‐sEV and Ctrl‐sEV groups. Each group contained three biological replicates. A) Correlation analysis for miRNA‐seq in the Cis‐sEV and Ctrl‐sEV groups. B) The different percentages of small RNA‐mapped reads in the Cis‐sEV and Ctrl‐sEV groups. C) A Venn diagram of the identified miRNAs between the Cis‐sEV and Ctrl‐sEV groups. D,E) Volcano plot analysis and heatmap clustering analysis of the 83 differentially expressed miRNAs, with 74 upregulated miRNAs and 9 downregulated miRNAs in the Cis‐sEV group. The color bar in (E) represents the relative expression levels. F)The expression of eight miRNAs listed in Table 1 was verified through RT‐qPCR; n = 3. All data are shown as the mean ± SD, with *p* < 0.001 marked as “***,” and “n.s” representing no significance. G) The target prediction analysis of the differentially abundant miRNAs, including target types and their coding capacity.

Small RNAs, particularly miRNAs, are crucial components of sEV and play a direct role in intercellular communication by regulating gene expression at the post‐transcriptional level.^[^ ^12^ ^]^ To detect the differentiation of miRNA molecular profiles between the Cis‐sEV and Ctrl‐sEV groups, a small RNA‐seq analysis was performed with three biological replicates for each group. First, PCA analysis revealed a clear discrepancy between the two groups (Figure S2A, Supporting Information). Additionally, correlation analysis indicated a strong correlation among replicates within each group (**Figure** **3**A). Notably, the miRNA composition in the sEV RNA categories was found to be more abundant in the Cis‐sEV group, when compared to the Ctrl‐sEV group (Figure 3B). 810 miRNAs were identified across all samples. Furthermore, the analysis revealed 799 miRNAs in the Cis‐sEV group and 400 miRNAs in the Ctrl‐sEV group, with 389 miRNAs being common in both groups (Figure 3C). The level of miRNAs in the Cis‐sEV group was markedly higher than that in the Ctrl‐sEV group, potentially offering valuable insights into the pathological changes associated with cochlear damage induced by cisplatin exposure. The expression levels of the top 40 miRNAs in both the Cis‐sEV and Ctrl‐sEV groups were presented in Figures S2B,C, Supporting Information, respectively. Furthermore, differential expression analysis revealed that 83 miRNAs were significantly altered between the two groups, of which 74 miRNAs were upregulated (including 7 novel miRNAs) and 9 miRNAs (including 3 novel miRNAs) were downregulated in the Cis‐sEV group compared to the Ctrl‐sEV group (Figure 3D,E). The seven novel miRNAs that exhibited differentially enriched expression in the Cis‐sEV group were cataloged in Table S1, Supporting Information. Additionally, the expression levels of numerous upregulated miRNAs were markedly increased in the Cis‐sEV group (Figure 3E). This includes several miRNAs known to regulate ROS reactions, inflammatory responses, and apoptosis, such as mmu‐miR‐100‐3p,^[^ ^13^ ^]^ mmu‐miR‐124‐3p,^[^ ^14^ ^]^ mmu‐miR‐129‐2‐3p,^[^ ^15^ ^]^ mmu‐miR‐140‐5p,^[^ ^16^ ^]^ mmu‐miR‐153‐3p,^[^ ^17^ ^]^ mmu‐miR‐15b‐5p,^[^ ^18^ ^]^ mmu‐miR‐17‐5p,^[^ ^19^ ^]^ mmu‐miR‐25‐3p,^[^ ^20^ ^]^ mmu‐miR‐339‐5p,^[^ ^21^ ^]^ mmu‐miR‐34a‐5p,^[^ ^22^ ^]^ and mmu‐miR‐370‐3p.^[^ ^23^ ^]^ Eight of these miRNAs, along with their potential functions and relevant references were summarized in **Table** **1**. Next, we validated the expression of the eight selected miRNAs using RT‐qPCR. The results revealed that mmu‐miR‐34a‐5p, mmu‐miR‐140‐5p, mmu‐miR‐15b‐5p, mmu‐miR‐25‐3p, and mmu‐miR‐339‐5p exhibited stable upregulation in Cis‐sEVs (Figure 3F), which was consistent with the sequencing data. These results reinforced the potential roles of the five miRNAs as signaling molecules or targets. From the 83 differentially expressed miRNAs, a total of 2537 target genes were predicted, enabling the investigation of their potential downstream effects. These predicted targets encompassed not only mRNAs but also numerous noncoding RNAs, including circRNAs and lncRNAs (Figure 3G), suggesting that these miRNAs likely played a wide range of regulatory functions within the cells. Subsequently, the targets of differentially expressed miRNAs merit further investigation.

**Table 1:** The functions and relevant references of eight enriched miRNAs in the Cis‐sEV group.

MiRNAs ID	Sequence (5′‐3′)	Functions and reference
mmu‐miR‐124‐3p	UAAGGCACGC GGUGAAUGCC	Positive regulation of apoptosis;^[^ ^14^ ^]^ positive regulation of inflammatory responses and oxidative stress;^[^ ^14a^ ^]^ positive regulation of carboplatin‐induced mitochondrial apoptosis;^[^ ^24^ ^]^ regulation of autophagy;^[^ ^25^ ^]^ negative regulation of cell population proliferation;^[^ ^26^ ^]^ miRNA‐mediated gene silencing.^[^ ^27^ ^]^
mmu‐miR‐140‐5p	CAGUGGUUUUA CCCUAUGGUAG	Regulation of oxidative stress and apoptosis;^[^ ^16^, ^28^ ^]^ regulation of inflammation;^[^ ^29^ ^]^ negative regulation of cell proliferation.^[^ ^30^ ^]^
mmu‐miR‐15b‐5p	UAGCAGCACAU CAUGGUUUACA	Regulation of apoptosis;^[^ ^17^, ^31^ ^]^ negative regulation of TGF‐beta receptor signaling pathway;^[^ ^32^ ^]^ negative regulation of mitotic cell cycle;^[^ ^33^ ^]^ positive regulation of mitochondrial fission.^[^ ^34^ ^]^
mmu‐miR‐17‐5p	CAAAGUGCUUAC AGUGCAGGUAG	Positive regulation of cytokine production associated with inflammatory responses;^[^ ^35^ ^]^ regulation of cell apoptotic processes;^[^ ^19^, ^36^ ^]^ negative regulation of cell proliferation;^[^ ^37^ ^]^ miRNA‐mediated gene silencing.^[^ ^38^ ^]^
mmu‐miR‐25‐3p	CAUUGCACUUG UCUCGGUCUGA	Regulation of cell apoptotic processes;^[^ ^20^, ^39^ ^]^ regulation of autophagy;^[^ ^40^ ^]^ miRNA‐mediated gene silencing.^[^ ^41^ ^]^
mmu‐miR‐339‐5p	UCCCUGUCCUCC AGGAGCUCACG	Regulation of cell apoptotic processes;^[^ ^42^ ^]^ regulation of inflammation;^[^ ^43^ ^]^ regulation of mitochondrial oxidative stress.^[^ ^44^
mmu‐miR‐34a‐5p	UGGCAGUGUCU UAGCUGGUUGU	Positive regulation of apoptotic processes and ROS accumulation;^[^ ^45^ ^]^ negative regulation of cell proliferation and intracellular signal transduction;^[^ ^46^ ^]^ miRNA‐mediated gene silencing;^[^ ^47^ ^]^ positive regulation of TGF‐beta production.^[^ ^48^ ^]^
mmu‐miR‐370‐3p	GCCUGCUGGGG UGGAACCUGGU	Regulation of apoptotic processes;^[^ ^23^ ^]^ positive regulation of NF‐κB signaling pathway;^[^ ^37^ ^]^ regulation of inflammatory responses.^[^ ^49^ ^]^

### 2.4 Differentially expressed miRNAs in Cis‐sEV Mediate Intercellular Communication and Damage in Cisplatin‐Induced Ototoxicity

**Figure 4:**
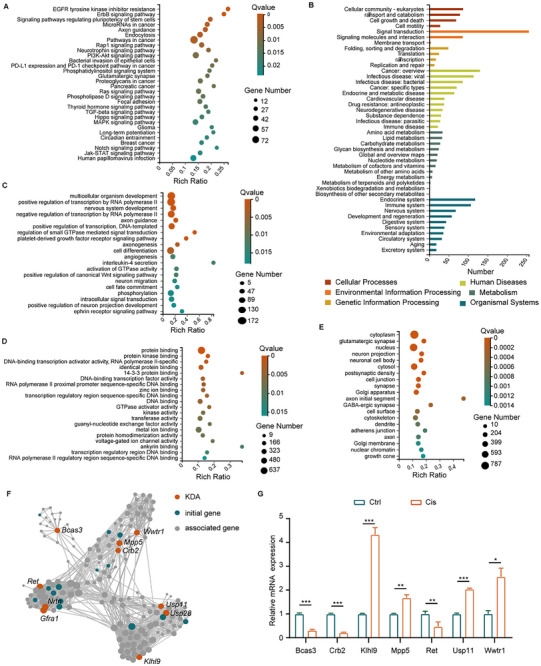
The downstream analysis of the targets regulated by 83 differentially expressed miRNAs in the Cis‐sEV group. A,B) KEGG enrichment and classification analysis of the targets indicated that many of these pathways were associated with apoptosis, inflammation, cell survival, and vesicle secretion. C–E) GO analysis was performed to categorize the biological processes (C), molecular functions (D), and cellular components (E). The color bar indicates the relative Q value. F,G) KDA network analysis with 10 genes in the key regulatory position labeled in the chart (F) and the verification of the 10 genes by RT‐PCR using samples of the cochlear explants (G). Data are presented as the mean ± SD; n = 3, with “*” for *p* < 0.05, “**” for *p* < 0.01, “***” for *p* < 0.001.

First, a KEGG pathway enrichment analysis was conducted for the 2537 targets of differentially expressed miRNAs in Cis‐sEV. This analysis revealed that 12 of the top 30 functional annotation pathways with the minimum Q values were related to critical biological processes such as cell survival, apoptosis, and inflammation. Notable pathways included ErbB, Rap1, neurotrophin, PI3K‐Akt, phosphatidylinositol, Ras, phospholipase D, focal adhesion, TGF‐beta, Hippo, MAPK, and Jak‐STAT signaling pathways (**Figure** **4**A). In addition, several pathways were related to vesicle secretion, including endocytosis, the phosphatidylinositol signaling system, and the phospholipase D signaling pathway (Figure 4A). Moreover, a KEGG pathway analysis across six categories was performed for the 2537 miRNA targets, with a particular focus on pathways related to cellular processes and environmental information processing. These pathways included signal transductions, signaling molecules and interactions, cellular community‐eukaryotes, transport and catabolism, and cell growth and death (Figure 4B), indicating that differentially expressed miRNAs in the Cis‐sEV group may participate in regulating characteristic cellular communication processes. Moreover, the enriched KEGG pathways related to the endocrine system, immune system, development, regeneration, and sensory system indicated that the miRNAs in the Cis‐sEV group probably play important roles in cross‐tissue regulatory following cisplatin‐induced ototoxicity (Figure 4B). GO analysis of biological processes and molecular functions showed that these miRNA targets were related to the process of multicellular organism development, nervous system development, axon guidance, regulation of small GTPase mediated signal transduction, positive regulation of canonical Wnt signaling pathway, and as well as participated in protein binding, protein kinase binding, DNA binding, and GTPase activator activity (Figure 4C,D), indicating these miRNAs may mediate important cellular function regulation in recipient cells. In addition, GO analysis of the cellular components showed that the predicted targets were largely enriched in the cytoplasm and nucleus (Figure 4E). The KDA was conducted to understand the genes that play major regulatory roles within the gene network of miRNA targets, thereby identifying 10 key driver genes (Figure 4F). Furthermore, these genes were verified in cochlear explants through RT‐qPCR (Figure 4G). The results indicated that seven genes (*Bcas3*, *Crb2*, *Klhl9*, *Mpp5*, *Ret*, *Usp11*, and *Wwtr1*) exhibited significant expression changes following cisplatin treatment. Notably, this study is the first to demonstrate the involvement of these genes in cisplatin ototoxicity, suggesting they may play a critical role in exacerbating cisplatin‐induced damage.

Overall, our findings imply that the differentially abundant miRNAs in the Cis‐sEV group likely play a vitally important role in intercellular communication related to ototoxic stimulation, and may mediate damage to the inner ear microenvironment by transferring signals involved in apoptosis, inflammation, and others.

### 2.5 Label‐Free Proteomic Profiling and Identification of Ctrl‐sEV and Cis‐sEV

**Figure 5:**
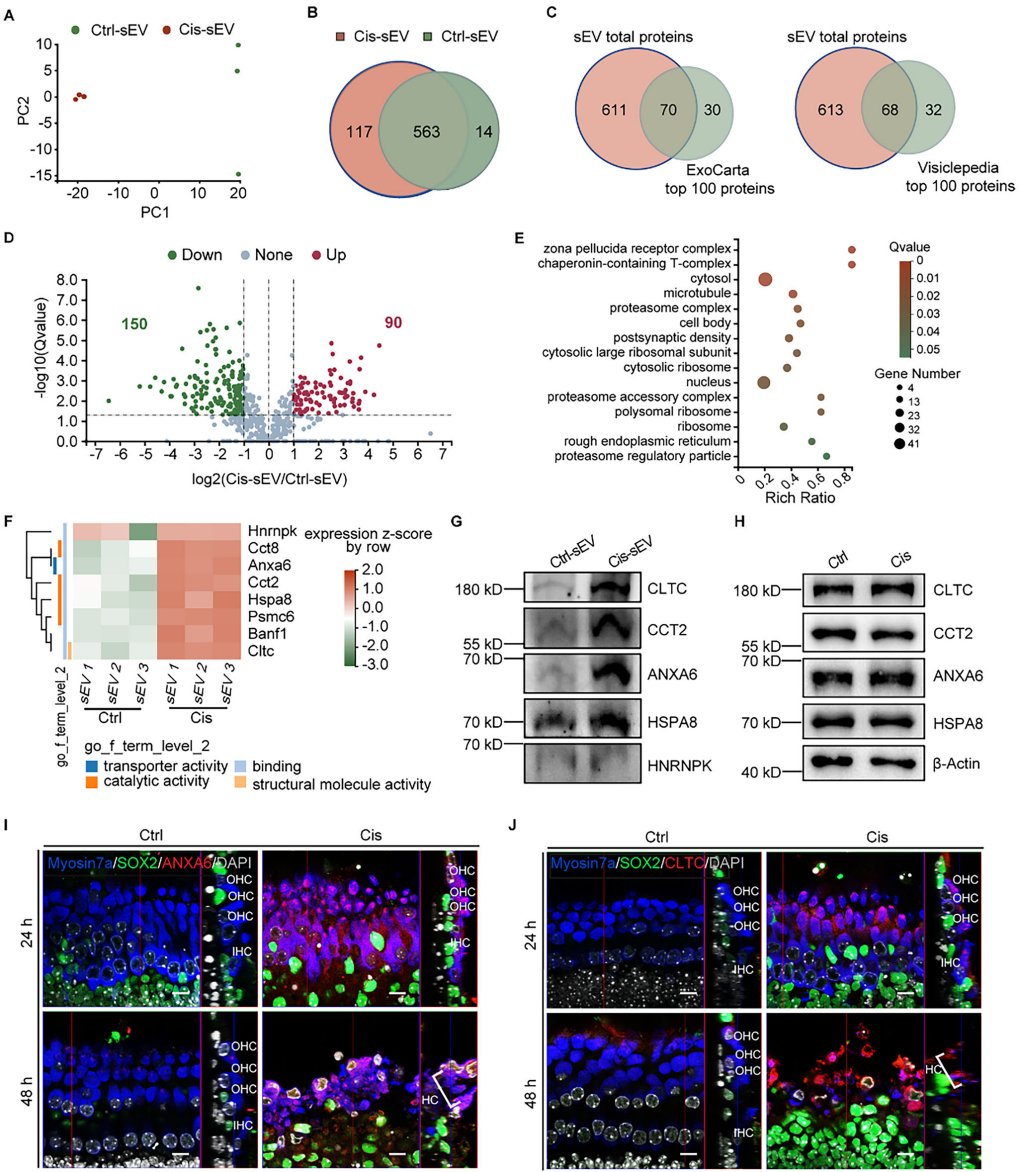
Proteome analysis and validation of the Cis‐sEV and Ctrl‐sEV groups. A) PCA analysis of proteins in the Cis‐sEV and Ctrl‐sEV groups. B) A Venn diagram indicating the unique and common proteins between Cis‐sEV and Ctrl‐sEV. C) A Venn diagram depicting the overlapping proteins among the total 694 proteins found in the sEVs and the top 100 sEV proteins from the ExoCarta and Vesiclepedia databases, respectively. D) Volcano plot analysis shows the 240 differentially expressed proteins, including 90 upregulated proteins and 150 downregulated proteins in the Cis‐sEV group. E) GO analysis of the cellular components for the 90 differentially enriched expression proteins. The color bar represents the relative Q value, and a Q value <0.05 indicates significant differences. F) Heat map analysis for the eight proteins associated with the apoptosis, autophagy, proteostasis, and inflammasome pathways. The color bar represents the relative expression level. G) Expression verification of CLTC, CCT2, ANXA6, HSPA8, and HNRNPK in the Ctrl‐sEV and Cis‐sEV derived from cochlear explant, normalized according to BCA protein quantification results. The experiment was repeated two times independently. H) The protein levels of CLTC, CCT2, ANXA6, and HSPA8 were compared in cisplatin‐treated cochlear explants and control explants after 48 h. β‐Actin served as the reference protein, and the experiment was conducted three times, independently. I,J) Fluorescence confocal microscopy images illustrate the expression patterns of ANXA6 and CLTC (red) in both control and 50 µM cisplatin‐treated cochlear explants at 24 h and 48 h. The right panels display partial longitudinal sections of the explants. Myosin7a (blue) marks cochlear HCs, SOX2 (green) marks cochlear supporting cells, and DAPI is the nuclear marker. Scale bars, 10 µm. OHC, outer hair cell; IHC, inner hair cell.

To identify the differentially expressed proteins in Cis‐sEV, a label‐free LC‐MS/MS analysis was conducted to investigate the proteome of both the Cis‐sEV and Ctrl‐sEV groups. The PCA analysis revealed a significant discrepancy between the two groups (**Figure** **5**A). There was a total of 694 proteins detected across the Cis‐sEV and Ctrl‐sEV groups, with 563 proteins overlapping between the two groups, as shown in the Venn diagram in Figure 5B. Also, the total proteins of the sEV showed overlaps 70 and 68 proteins with the top 100 sEV proteins in the two EV databases of ExoCarta (exocarta.org/Archive/ExoCarta_top100_protein_details_5.txt) and Vesiclepedia (Vesiclepedia: Extracellular vesicle Markers (microvesicles.org)), respectively (Figure 5C), indicating a high degree of purity of the sEV in our study. Among the 563 common proteins, 150 proteins were significantly decreased, and 90 proteins were increased dramatically in Cis‐sEV compared to Ctrl‐sEV, as shown in the volcano plot and heat map clustering analysis (Figure 5D, and Figure S3A,B, Supporting Information). The enriched GO cellular components for these 90 upregulated proteins in Cis‐sEV indicated the intracellular origins of EVs, including the cytosol, microtubule, cell body, ribosome, nucleus, endoplasmic reticulum, chaperonin‐containing T‐complex, and several proteasome complexes, which may be associated with the disrupted proteostasis induced by cisplatin (Figure 5E). It is well known that maintaining cellular homeostasis is crucial in response to injurious stimuli. We further selected eight proteins related to apoptosis, autophagy, proteostasis, and inflammasome pathways for further analysis, as shown in Figure 5F. The functions and relevant references of these eight differentially abundant proteins were listed in **Table** **2**.

**Table 2:** The functions and relevant references of eight differentially abundant proteins in the Cis‐sEV group.

Protein ID	Symbol	Functions and references
tr|B2M1R6|B2M1R6_MOUSE	HNRNPK	Involved in the DNA damage response;^[^ ^50^ ^]^ LC3‐conjugation loaded into EVs;^[^ ^51^ ^]^ potential modulator of exosomal RNA sorting;^[^ ^52^ ^]^ DNA/RNA‐binding protein and regulation of gene transcription;^[^ ^53^ ^]^ regulation of cancer progression.^[^ ^54^ ^]^
tr|H3BL49|H3BL49_MOUSE	CCT8	Regulation of cell cycle, proliferation, invasion, and metastasis;^[^ ^55^ ^]^ control of proteostasis as a chaperonin;^[^ ^56^ ^]^ cell‐specific transcriptional response to heat shock in the mouse utricle epithelium.^[^ ^57^ ^]^
tr|Q99JX6|Q99JX6_MOUSE	ANAX6	Regulation of autophagy;^[^ ^58^ ^]^ regulation through ANXA6‐containing extracellular vesicles, such as mediating drug resistance;^[^ ^58^, ^59^ ^]^ metabolic regulation.^[^ ^60^ ^]^
sp|P80314|TCPB_MOUSE	CCT2	An aggrephagy receptor for clearance of solid protein aggregates;^[^ ^9^, ^61^ ^]^ enrichment in extracellular vesicles and mediation of signaling communication;^[^ ^62^ ^]^ regulation of cell cycle.^[^ ^63^ ^]^
tr|Q3UDS0|Q3UDS0_MOUSE	HSPA8	A crucial molecular regulator of chaperone‐mediated autophagy;^[^ ^64^ ^]^ a pan‐EV protein marker;^[^ ^65^ ^]^ regulation of inflammasome pathways.^[^ ^66^ ^]^
sp|P62334|PRS10_MOUSE	PSMC6	Regulation of apoptosis;^[^ ^67^ ^]^ participation in ubiquinone metabolism.^[^ ^68^ ^]^
sp|O54962|BAF_MOUSE	BANF1	Modulates DNA double‐strand break repair pathways;^[^ ^69^ ^]^ controls the DNA damage response to oxidative stress or autoinflammation;^[^ ^70^ ^]^ repair of nuclear ruptures.^[^ ^71^ ^]^
sp|Q68FD5|CLH1_MOUSE	CLTC	Regulation of the uptake of exosomes by cells;^[^ ^72^ ^]^ mediating interactions between autophagic vesicles and the Coxiella‐containing vacuole;^[^ ^73^ ^]^ regulation of apoptosis;^[^ ^74^ ^]^ the initiation of the endosomal ESCRT degradation pathway;^[^ ^75^ ^]^ enhancing endosomal trafficking and blocking autophagic degradation.^[^ ^76^ ^]^

Five proteins of interest, which were involved in endocytosis, protein homeostasis, and autophagy, were selected for validation by immunoblotting. Notably, CLTC, CCT2, ANXA6, and HSPA8 exhibited increased abundance in Cis‐sEV, while heterogeneous nuclear ribonucleoprotein K (HNRNPK) showed no significant difference between Cis‐sEV and Ctrl‐sEV (Figure 5G). Moreover, CLTC, CCT2, ANXA6, and HSPA8 were also ranked within the top 100 sEV proteins according to both ExoCarta and Vesiclepedia databases. Interestingly, these four proteins did not significantly differ in cochlear explant lysates (Figure 5H). This suggested these proteins were potentially integral to specific intercellular communication related to cisplatin‐induced ototoxicity through sEV. To further investigate the spatially orchestrated pathology, we conducted fluorescence expression analysis of ANXA6 and CLTC proteins that were specifically enriched in Cis‐sEV. The results showed that, compared to the control group, both ANXA6 and CLTC proteins were enriched and exclusively co‐localized in HCs after cisplatin‐induced damage, regardless of whether the drug was treated for 24 or 48 h (Figure 5I,J). Furthermore, no significant expression of ANXA6 and CLTC was observed in the SOX2‐labeled supporting cells or any other cells beneath the HCs in the cochlear basilar membrane explants, as shown in the longitudinal sections of confocal images (Figure 5I,J). This finding suggests that the cisplatin‐damaged HC population is the primary parent cell for Cis‐sEV in cochlear explants.

### 2.6 CLTC Expression in sEV as a Potential Biomarker for Cisplatin‐Induced Ototoxicity

**Figure 6:**
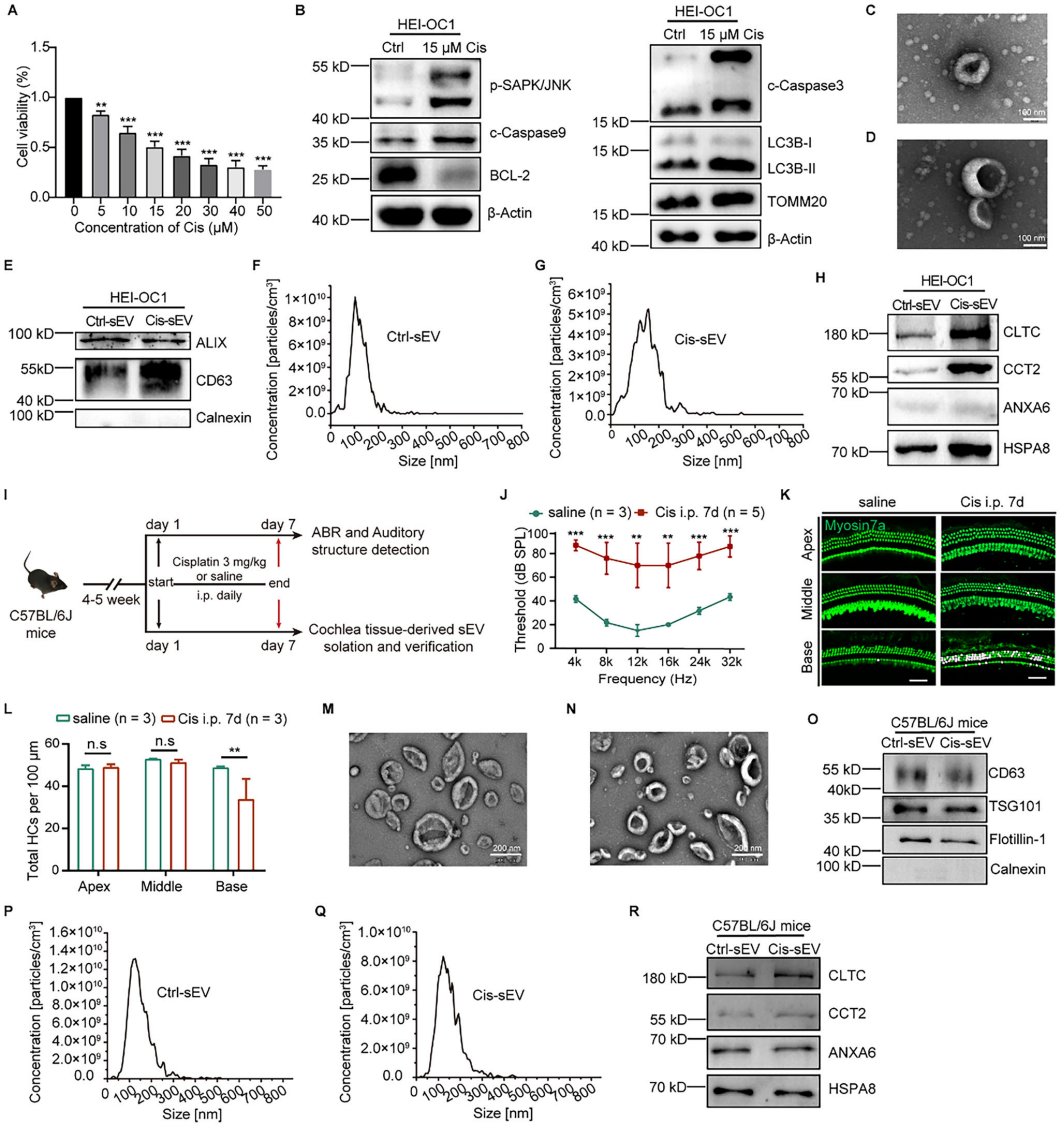
Verification of protein expressions in Cis‐sEV using both in vitro and in vivo ototoxicity models. A) Viability of HEI‐OC1 cells treated with various concentrations of cisplatin for 24 h. n = 3. All results showed as the mean ± SD, with “**” for *p* < 0.01, and “***” for *p* < 0.001. B) Western blot analysis in the HEI‐OC1 model confirmed the expression of damage‐associated molecules after 15 µM cisplatin treatment for 48 h. β‐Actin serves as the reference protein. C,D) TEM analysis of the morphology of Cis‐sEV and Ctrl‐sEV in the HEI‐OC1 damage model. Scale bar = 100 nm. E) Both positive and negative markers (Alix, CD63, Calnexin) were analyzed by western blotting for Ctrl‐sEV and Cis‐sEV in the cisplatin‐induced HEI‐OC1 model, normalized according to BCA protein quantification results. F,G) NTA analysis of Ctrl‐sEV and Cis‐sEV in the HEI‐OC1 damage model. H) Validation of CLTC, CCT2, ANXA6, and HSPA8 expression in sEV within the HEI‐OC1 damage model, normalized according to BCA protein quantification results. The experiment was performed independently and repeated three times. I) A flowchart depicts the mouse model for cisplatin‐induced ototoxicity. For a week, the 4–5‐week‐old C57BL/6J mice received daily i.p. injections of either cisplatin (3 mg kg^−1^ day^−1^) or an equivalent amount of saline. J) The analysis of the ABR threshold was performed on mice exposed to either cisplatin or saline for 7 days. Saline group: n = 3; Cis group: n = 5. The “n'” indicates the number of mice included in the statistics analysis. Results are presented as mean ± SD, with *p* < 0.01 indicated as “**” and *p* < 0.001 indicated as “***.” K) Representative images of HCs in the mouse cochlea treated with saline or cisplatin, including the apical, middle, and basal turns. Myosin7a (green) labels the cochlear HCs, while white “*” indicates the absent HCs, scale bar = 50 µm. L) Statistical assessment of the counts of HCs per 100 µm of the basilar membrane in every turn of mouse cochlea following treatment with saline and cisplatin. The “n” represents the number of mice. Results are shown as mean ± SD, with *p* < 0.01 marked as “**,” and “n.s” indicating the no signification. M,N) TEM characterization of Ctrl‐sEV and Cis‐sEV derived from mouse cochlear tissues, scale bar = 200 nm. O) Verification of protein markers in Ctrl‐sEV and Cis‐sEV derived from mouse cochlear tissues; normalized according to BCA protein quantification results. P,Q) NTA analysis of Ctrl‐sEV and Cis‐sEV derived from mouse cochlear tissues. R) Expression verification of CLTC, CCT2, ANXA6, and HSPA8 in Ctrl‐sEV and Cis‐sEV derived from mouse cochlear tissues, normalized according to BCA protein quantification results. The experiment was independently repeated three times.

We also developed an in vitro model using HEI‐OC1 cells to further ascertain that these four specific proteins were responsive to cisplatin‐induced ototoxicity and were released in sEV. 15 µM cisplatin was used to establish the in vitro model, with cell viability remaining at ≈50% following 24 h of treatment (**Figure** **6**A). The expression pattern of injury markers detected through immunoblotting, including upregulation of phosphorylated SAPK/JNK, cleaved‐Caspase9, cleaved‐Caspase3, LC3B, and TOMM20, as well as Bcl‐2 downregulation in cisplatin treatment at 48 h, confirmed the successful construction of the in vitro damage model (Figure 6B). We then isolated the sEV from the cell culture medium and characterized them by TEM, western blotting, and NTA (Figure 6C–G), which indicated the sEV was successfully obtained. Importantly, the three proteins of interest – CLTC, CCT2, and HSPA8 – were also abundantly expressed in Cis‐sEV in the cisplatin‐induced HEI‐OC1 model, while ANXA6 was not (Figure 6H). Also, none of the four proteins exhibited differentially expressed in the cisplatin‐treated cells compared to untreated cells (Figure S4, Supporting Information).

Furthermore, to further validate the findings, we constructed an in vivo cisplatin‐induced ototoxicity mouse model by administering cisplatin at a dosage of 3 mg kg^−1^ day^−1^ for seven consecutive days (Figure 6I). The auditory brainstem response (ABR) test results demonstrated a significant increase in hearing threshold across all frequencies, indicating near‐total deafness in the mice (Figure 6J). Histological examination of the cochlear structure revealed a noticeable loss of HCs starting from the basal turn (Figure 6K,L). These results identified the successful establishment of an acute cisplatin‐induced ototoxicity model. The observed spatial dissociation between focal HCs loss (basal turn‐specific) and global ABR threshold shifts (40‐55 dB across frequencies) is probably due to multilayered damage patterns of synaptic/neural dysfunction over HCs death in early‐stage ototoxicity.^[^ ^77^ ^]^ Subsequently, we extracted sEV derived from mouse cochlear tissue using enzyme digestion with collagenase D and DNase І, as reported earlier by our laboratory.^[^ ^9c^ ^]^ TEM analysis confirmed that both Ctrl‐sEV and Cis‐sEV exhibited typical cup‐shaped morphology (Figure 6M,N). Furthermore, both types of sEVs expressed specific marker proteins CD63, TSG101, and Flotillin‐1 but lacked the endoplasmic reticulum marker protein Calnexin (Figure 6O). Additionally, both Ctrl‐sEV and Cis‐sEV were characterized by NTA, which showed that their particle sizes were predominantly in the range of 200 nm (Figure 6P,Q). We then examined the protein expression levels of CLTC, CCT2, ANXA6, and HSPA8 in Ctrl‐sEV and Cis‐sEV derived from mouse cochlear tissue. Notably, CLTC protein was significantly upregulated in Cis‐sEV, consistent with our previous findings in the in vitro cochlear explant and HEI‐OC1 injury models (Figure 6R). These results indicated that CLTC protein expression is stably increased in Cis‐sEV across various cisplatin injury models, indicating its potential as a biomarker or therapeutic target for mitigating the ototoxic effects of cisplatin.

## 3 Discussion

To address the structural inaccessibility of the adult murine osseous labyrinth,^[^ ^9b^ ^]^ we established a neonatal cochlear explant‐based ototoxic stress model optimized for sEV isolation. For comprehensive molecular characterization, we implemented complementary high‐resolution profiling workflows: sEV miRNA analysis via small RNA sequencing, coupled with deep proteome interrogation using label‐free quantitative LC‐MS/MS. This dual‐omics approach ensures sensitive detection of both regulatory miRNAs and functional proteins within cochlea‐derived sEVs.

The miRNA profile of Cis‐sEV exhibits marked fluctuations, with 83 differentially expressed miRNAs identified compared to Ctrl‐sEV (Figure 3D,E). Notably, several auditory function‐associated miRNAs were dysregulated, including miR‐182‐3p and miR‐96‐3p from the miR‐183 family which are essential regulators for inner ear HCs development and survival.^[^ ^78^ ^]^ Cis‐sEV particularly demonstrated significant upregulation of miRNA‐34a‐5p, which is implicated in cochlear pathology that mediates HC apoptosis and ROS accumulation through miR‐34a/SIRT1 axis activation in the aged cochlea.^[^ ^79^ ^]^ Of particular significance, the other four enriched miRNAs (miR‐140‐5p, miR‐15b‐5p, miR‐25‐3p, and miR‐339‐5p), experimentally validated in Cis‐sEV, show direct associations with three key ototoxic mechanisms: ROS amplification, inflammatory cascades, and programmed cell death (Table 1). These molecular signatures imply that Cis‐sEV miRNAs may orchestrate hearing loss progression through multimodal pathway regulation, potentially modulating HC stress adaptation, survival signaling, and disruption of cochlear homeostasis.

Our multi‐omics integration reveals coordinated miRNAs targeting core pathological pathways in cisplatin ototoxicity — particularly cellular survival networks, apoptotic cascades, and inflammatory signaling. Cross‐tissue regulatory potential is evidenced by KEGG enrichment across endocrine, immune, and sensory systems, suggesting Cis‐sEV miRNAs may orchestrate cochlear damage through both local microenvironment disruption (e.g., HCs loss) and systemic pathway crosstalk. Notably, functional network analysis identified seven previously unreported mediators of cisplatin ototoxicity (Figure 4G), whose cisplatin‐induced dysregulation may implicate autophagy modulation,^[^ ^80^ ^]^ cell apoptosis,^[^ ^81^ ^]^ DNA damage,^[^ ^82^ ^]^ protein homeostasis, cell survival, and neuron activity.^[^ ^83^ ^]^ BCAS3 downregulation may destabilize p53 by impairing CRL4A‐mediated ubiquitination,^[^ ^81^ ^]^ which could sensitize cells to apoptotic stress. Additionally, the overexpression of USP11 may enhance autophagic flux, potentially increasing vulnerability to ferroptosis.^[^ ^80b^ ^]^ Furthermore, Wwtr1 dysregulation may point to Hippo pathway involvement in cochlear homeostatic collapse.^[^ ^84^ ^]^ These first‐reported cisplatin ototoxicity‐associated mediators, functionally linked through protein homeostasis and stress‐response signaling, represent novel therapeutic entry points for intercepting multi‐level ototoxic damage.

Proteomic profiling of cochlear‐derived sEVs reveals cisplatin‐induced proteome remodeling, characterized by depletion of cochlear structural markers (OC90/TECTA/TECTB; Figure S3B, Supporting Information) and selective enrichment of 90 proteins in Cis‐sEV. We validated four differentially expressed proteins (CLTC, HSPA8, ANXA6, and CCT2) that were specifically overexpressed in Cis‐sEV, consistent with LC‐MS/MS analysis. CLTC, a scaffold protein in clathrin‐coated vesicles, could potentially amplify proteostatic crisis through vesicle trafficking hijacking.^[^ ^72^, ^85^ ^]^ HSPA8 (also known as HSC70) operates as a dual‐function mediator. While maintaining cochlear proteostasis as a molecular chaperone under basal conditions, its sEV hyper‐loading may override recipient cells' autophagic capacity through chaperone‐mediated pathway saturation.^[^ ^65^, ^86^ ^]^ ANXA6, a Ca^2+^‐dependent annexin protein, recapitulates chemoresistance‐associated EV export in breast cancer and can promote breast tumor metastasis and induce autophagy.^[^ ^58a^ ^]^ CCT2 is essential for cellular protein homeostasis and could extensively mirror tumor‐derived EV signatures.^[^ ^9^, ^62^ ^]^ These effectors collectively establish Cis‐sEV as cochlear stress signalosomes that orchestrate intercellular damage cascades in cisplatin stress. Crucially, two validated Cis‐sEV‐specific overexpression effectors (CLTC, ANXA6) exhibited HC‐restricted vesicle loading — their sEV‐specific overexpression correlated with spatial co‐localization in cisplatin‐damaged HCs while remaining unaltered in supporting cells or whole‐tissue lysates (Figure 5H–J). This compartmentalized packaging not only suggests the HC population damaged by cisplatin is probably the primary donor of Cis‐sEVs, which is consistent with the fact that cochlear HCs are the main sites of damage for cisplatin in the organ of Corti,^[^ ^87^ ^]^ but also suggests HCs employ polarized sEV biogenesis as cochlear stress signalosomes to disseminate ototoxic signals. These findings offer valuable insights into the biology of cochlea‐derived sEVs in reaction to cisplatin, which may enhance our knowledge of ototoxicity and help identify potential targets for therapeutic intervention.

The emerging paradigm of sEV‐based liquid biopsy has opened new frontiers in ototoxicity monitoring.^[^ ^88^ ^]^ Our cross‐model validation identified CLTC as a conserved Cis‐sEV signature protein, with sustained upregulation in cochlear explants, HEI‐OC1 cells, and rodent models. The discrepancy in the expression of the other three candidates between in vitro and in vivo models may be due to three factors: (1) cell type heterogeneity between immortalized HEI‐OC1 progenitors and native cochlear multicellular niches, (2) absence of systemic microenvironmental modulators (e.g., inflammatory signaling) in vitro that govern EV biogenesis in vivo, and (3) stress‐dependent EV packaging reflecting distinct cisplatin injury dynamics. The multilayered consistency positions CLTC as a translational pivot for non‐invasive ototoxicity assessment, particularly when integrated with breakthrough technologies like the immunomagnetic nano pom‐pom platform. The latter's success in capturing perilymph sEVs from SNHL patients samples less than 5 µL demonstrates clinical‐grade feasibility — a critical advancement considering the cochlea's microliter‐scale fluid compartments.^[^ ^89^ ^]^ Compared to conventional audiometry, sEV biomarker quantification provides clear advantages, including objective readouts that eliminate patient response variability and molecular granularity that detects subclinical injury before threshold shifts. However, extensive clinical studies and validations are required to substantiate this possibility. Nonetheless, the clinical implementation faces two primary challenges: (1) temporal specificity — distinguishing cisplatin‐specific signatures from age‐related and noise‐induced hearing loss backgrounds; and (2) technical standardization — establishing consensus protocols for sEV isolation from ultra‐low volume perilymph (≤10 µL) and CLTC detection thresholds that are correlated with functional hearing loss.

Overall, our ongoing efforts focus on establishing a diagnostic‐therapeutic nexus, including validating clinical predictive value of CLTC through longitudinal trials and exploring strategies targeting proteins and miRNAs for early ototoxicity interception. Significant effort is required to translate these findings into clinical applications that improve the quality of life for cancer patients.

## 4 Experimental Section

### Animals

P3 ICR mice (Strain No. N000293) were purchased from GemPharmatech Company (Nanjing, China). C57BL/6J mice aged 4–5 weeks were obtained from Jiangsu Qinglongshan Biotechnology Co.L TD (Danyang, China). The study included both male and female mice. All animal‐related procedures were permitted by the Animal Care and Welfare Committee of Southeast University with the approval number of No. 20 210 503 031 and were followed by the National Institute of Health's Guide for the Care and Use of Laboratory Animals. Every effort was made to minimize the suffering of animals involved in the experiments and to use as few animals as possible.

### Explant Culture, and the Ex Vivo Cisplatin‐Induced Ototoxicity Model

The dissociation and culture of P3 cochlear explants were performed in a sterile environment with sterile reagents. In brief, P3 ICR mice were sacrificed via cervical dislocation, and their cochlear sensory epithelium was dissected in cold HBSS at pH 7.4. The cochlear bony labyrinth was carefully exposed under a microscope to remove the spiral ganglion and other epithelial tissues. The unbroken cochlear basement membrane tissue was then transferred onto a coverslip using Cell‐Tak (Corning, 354 240), placed into a four‐well culture dish (Corning, 627 170), and cultured with 2 mL DMEM/F12 medium (Gibco, 11330‐032) added with 10% (v/v) fetal bovine serum (FBS; PAN biotech, ST30‐3302) and 0.05% (v/v) ampicillin at 37 °C with 5% CO_2_. After being allowed to recover overnight, the explants were washed twice using phosphate‐buffered saline (PBS). 50 µM cisplatin (Sigma, P4394) or an equal volume of PBS was treated for 48 h to induce ototoxicity *ex vivo*, as previously reported. The culture medium was collected and replaced every 24 h.

### Cell Culture, and In Vitro Cisplatin‐Induced Ototoxicity Model

For the in vitro model of cisplatin‐induced ototoxicity, HEI‐OC1 cells were employed. These cells were cultured in high‐glucose DMEM (Vivacell, C3103‐0500), which contained 10% (v/v) FBS and 0.05% (v/v) ampicillin to foster optimal growth conditions. When the HEI‐OC1 cells reached 70%–80% confluence after 12 h, they were treated with cisplatin or an equivalent volume of PBS for 48 h to induce ototoxicity in vitro. The medium was collected every 24 h to isolate sEV.

### Cell Counting Kit‐8 Assay

HEI‐OC1 cells were cultured in 96‐well plates and exposed to different concentrations of cisplatin (including 0, 5 10, 15, 20, 30, and 40 µM) for a duration of 24 h. The cell Counting Kit reagent (Beyotime, C0038) was used to assess the cell viability, which was diluted to 10% in DMEM medium, followed by a 30‐min incubation at 37 °C. Wells without cells served as the blanks. The optical density (OD) values at 450 nm were recorded by a microplate photometer (Thermo Fisher Scientific, Multiskan FC). Cell viability was calculated as follows: Cell viability (%) = [(ODsample‐ODblank) / (ODcontrol‐ODblank)] × 100%.

### Animal Model of Cisplatin‐Induced Ototoxicity

In vivo, cisplatin treatment involved intraperitoneal (i.p.) injections of 4–5‐week‐old C57BL/6J mice with cisplatin (3 mg kg^−1^ body weight), diluted in 0.9% physiologic saline at a concentration of 0.5 mg mL^−1^, once daily for subcutaneously 7 days. The control group received equivalent volumes of 0.9% physiologic saline via i.p. injection, calculated as 0.6 mL per 100 g of body weight. Both sexes of C57BL/6J mice were randomly divided into two groups. Audiological measurements and cochlear tissue collection were performed 24 h after the final cisplatin administration (Day 8), without recovery period intervention. This acute time point selection aligns with prior characterization studies of cisplatin‐induced ototoxicity.^[^ ^77^ ^]^

### Immunostaining, HC Counting, and Mito‐Sox Staining

For immunostaining, the cochlea samples were fixed at room temperature for 1 h using 4% (v/v) paraformaldehyde. Next, the samples were washed with 1% PBST buffer (1% [v/v] Triton X‐100 in PBS) for ≈15 min and then blocked at room temperature for 1 h to prevent non‐specific antibody binding. After that, anti‐Myosin7a (Proteus Biosciences, 25–6790, 1:1000; DSHB, 138‐1, 1:400), anti‐Sox2 (R&D systems, AF2018, 1:200), anti‐Cleaved Caspase‐3 (Cell Signaling Technology, #9661, 1:400), anti‐CLTC (Abcam, ab21679, 1:300), and anti‐ANXA6 (Proteintech, 12542‐1‐AP, 1:100) primary antibodies were used and incubated overnight at 4 °C. After three additional washes with 1% PBST, the samples were treated with the Alexa Fluor‐conjugated secondary antibody (Invitrogen, A32766, A32731, A31570, A‐11055, A‐31571, 1:400) for 1 h at room temperature. Following three additional washes, the samples were infiltrated in Dako fluorescence mounting medium (DAKO, S3023) and immobilized with nail polish. Last, a laser scanning confocal microscope (Zeiss, LSM700) was used to image the samples. As the representative images, every turn of the cochlea was randomly captured in two 20 × low‐magnification confocal images. Then, the number of total HCs was counted in each turn using ImageJ (NIH Bethesda, MD), and the data were averaged per 100 µm.

The Mito‐Sox red mitochondrial superoxide indicator kit (Thermo Fisher Scientific, M36008) was used to detect the accumulation of mitochondrial ROS, following the manufacturer's regulations. Briefly, 5 µM Mito‐Sox was incubated with the cultured explants at 37 °C for 10 min. After three washes using PBS, the explants were conducted immunostaining in the dark as described above.

### Western Blotting

In brief, tissue and cell samples were lysed using 1 × RIPA lysis buffer (Epizyme, PC‐101), while sEV samples were lysed in 10 × RIPA lysis buffer (Millipore, 20–188). Both of them were supplemented with 1 × complete protease inhibitor cocktail (Roche, 0 469 313 2001). Protein concentrations were measured using a BCA protein assay kit (Bryotime Biotechnology, P0010). sEV lysates containing 10 µg total protein (derived from cochlea explants and HEI‐OC1 cells) or 5 µg total protein (derived from adult mice cochlea) were loaded per lane. Meanwhile, 20 µg tissue and cell lysates were separated via 7.5%–15% SDS‐polyacrylamide gel electrophoresis (PAGE) (Thermo Fisher Scientific) and then transferred to polyvinylidene difluoride membrane (Millipore, IPVH00010) with a 0.45 µm pore size. After blocking with 5% (w/v) skim milk diluted in TBST buffer at room temperature for 1 to 3 h, the blots were directly incubated overnight with primary antibodies at 4 °C. TBST buffer was prepared using 1 × Tris‐base saline solution at pH 7.4 with 0.1% (v/v) Tween‐20. Following washes, HRP‐conjugated secondary antibodies (Abmart, M21001s and M21002s, 1:4000) were applied for 1 h at room temperature. Last, the blots were photographed using a SuperSignal West Pico PLUS Chemiluminescent Substrate (Thermo Fisher Scientific, 34 580) and captured with a Tanon 2500R system (Tanon, China).

Antibodies against the ROS‐related protein TOMM20 (Proteintech, 11802‐1‐AP, 1:20 000), the inflammation‐related protein phosphorylated (p‐)SAPK/JNK (Cell Signaling Technology, #4668, 1:1000), and the pro‐apoptosis protein markers cleaved‐Caspase9 (Cell Signaling Technology, #9502, 1:1000) and cleaved‐Caspase3 (Cell Signaling Technology, #9661, 1:1000) were used for the analysis of the cochlear explants by western blotting, with β‐Actin (Abmart, P30002M, 1:10 000) serving as the internal reference. Moreover, Bcl‐2 (Santa Cruz Technology, sc‐7382, 1:200), and LC3 (Cell Signaling Technology, #4108, 1:1000) were used in HEI‐OC1 lysate. In addition, the EV‐positive markers CD63 (Abcam, ab217345, 1:1000), TSG101 (Abcam, ab125011, 1:1000), Flotillin‐1 (Cell Signaling Technology, #3253S, 1:1000), and Alix (Santa Cruz Technology, sc‐53540, 1:200) and the negative marker Calnexin (endoplasmic reticulum marker; Abcam, ab22595, 1:1000) were used for the analysis of the sEV samples. Antibodies against CLTC (Abcam, ab21679, 1:1000), CCT2 (Abcam, ab92746, 1:1000), ANXA6 (Proteintech, 12542‐1‐AP, 1:1000), HSPA8 (Santa Cruz Technology, sc‐7298, 1:200), and HNRNPK (Santa Cruz Technology, sc‐28380, 1:200) were also used in this study.

### RNA Extraction and Real‐Time Quantitative PCR

Total RNA from cultured cochlear explants was extracted using the conventional TRIzol reagent (Invitrogen, 15 596 026). To isolate total RNA from sEV, the MiRNeasy Micro kit (QIAGEN, 217 084) was employed, following the manufacturer's instructions. The RNA's concentration and quality were assessed on a nano‐500 micro‐spectrophotometer (Allsheng, China). A cDNA Synthesis Kit (Vazyme, R312) was employed to convert the extracted tissue RNA into complementary DNA (cDNA), while a miRNA first Strand cDNA Synthesis Kit (Vazyme, MR101) was used to synthesize miRNA cDNA by stem‐loop. Subsequently, RT‐qPCR was conducted with the universal SYBR qPCR Master Mix kit (Vazyme, Q712) and the miRNA Unimodal SYBR qPCR Master Mix Kit (Vazyme, MQ102) on an Applied Biosystems QuantStudio 3 (ABI, China) instrument. All RT‐qPCR primers for mRNA can be found in Table S2, Supporting Information. All primer sequences used for miRNA first Strand cDNA synthesis are shown in Table S3, Supporting Information, and those used for miRNA RT‐qPCR are shown in Table S4, Supporting Information. The housekeeping gene *β‐Actin* and the U6 gene were used to normalize the expression of mRNA and miRNA, respectively. Finally, the relative mRNA and miRNA expression were analyzed by comparative Ct value and calculated using the 2^‐△△Ct^ method.

### sEV Isolation and Purification from the Culture Medium

The sEV‐free FBS medium was used to culture the cochlear explants and HEI‐OC1 cells with PBS or cisplatin treatment for 48 h, and the conditional culture medium was collected and stored at 4 °C. sEVs were isolated from the supernatant within 1 week. In one experiment for characterizing sEV and verifying the expression of miRNAs and proteins, the culture medium from 120 cochlear explants was used for each group to isolate sEV. Additionally, 100 mL of culture supernatant from HEI‐OC1 cells was used in one experiment to isolate sEV. The process of sEV isolation and purification is shown in Figure 2A. A series of sequential centrifugation steps were employed to remove cells and cellular debris, including centrifugation at 600 × *g* for 10 min and 2000 × *g* for 20 min at 4 °C. To eliminate large vesicles, the supernatant was subsequently filtered through a 0.22 µm filter (Millipore, SLGPR33RB) and then centrifugated at 16 500 × *g* at 4 °C for 20 min. sEVs were concentrated with a 100 kD molecular weight cut‐off ultrafiltration device (Pall, Shanghai, China) and precipitated via ultracentrifugation at 110 000 × *g* for 2 h at 4 °C using a Himac CP‐90NX ultracentrifuge (Himac, Japan). After discarding the supernatant, the pellet was resuspended and ultracentrifuged at 110 000 × *g* for an additional 2 h to further eliminate any remaining contaminants. Last, the purified sEV pellet was re‐suspended in 200 µl D‐PBS and then stored at either 4 or −80 °C for further use.

sEV‐free FBS was used to eliminate the effect of serum‐derived sEV. This was obtained by ultra‐centrifuging the FBS at 170 000 × *g* overnight (more than 12 h) at 4 °C, and then collecting two‐thirds of the supernatant and filtrating through a 0.22 µm filter on a sterile and clean bench.

### sEV Isolation and Purification from Cochleae Tissue

The control and cisplatin‐treated mice cochlea tissue was dissected in the cold DPBS and then placed in 35 mm cell culture dishes (Greiner, 627 160) containing 1 mL cold DMEM (Vivacell, C3103‐0500). Each group contained 24 mice in one experiment, with their cochlear tissues divided into three dishes. Next, the bony shell of the cochlea is carefully crushed using tweezers to expose tissues, such as the basilar membrane, spiral ligament, modiolus, and stria vascularis. Subsequently, the exposed tissues were cut into small pieces using anatomical scissors. To each dish containing tissue, 3U Collagenase D and 40U DNase I were added uniformly, followed by a digestion period at 37 °C for 45 min. Digestion was immediately halted by placing the tissues on ice. The digested tissue was filtered using a 0.45 µm cell strainer to discard the bony shell and undigested tissue fragments. The filtrate, which contains sEV derived from the cochlear tissue, was then isolated and purified. The procedure was similar to that used for isolating sEV from a culture medium, involving centrifugation at 600 × *g* for 10 min and 2000 × *g* for 20 min, followed by filtering through a 0.22 µm filter (Millipore, SLGPR33RB) and then centrifugated at 16 500 × *g* for 20 min. Afterward, the supernatant was ultracentrifugation using a Himac CP‐90NX ultracentrifuge (Himac, Japan) at 4 °C, at 110 000 × *g* for two rounds, each lasting for 1.5 h. The purified sEVs were collected and stored at either 4 or −80 °C for further use.

### Nanoparticle Tracking Analysis and Transmission Electron Microscopy Analysis

For NTA, the sEV samples were appropriately diluted in PBS, and more than 6 detect positions per biological sample were analyzed. The Zeta View system (Particle Metrix, Meerbusch, Germany) was then utilized to evaluate the size distribution and particle concentration of the sEV, using the ZetaView version 8.05.14 SP7 software with 520 nm laser and sensitivity setting 80.0. Before the measurement, the instrument was calibrated in line with standard operating procedures. For TEM, the sEV sample was dropped onto a copper mesh and then negative stained with a uranyl acetate solution. After drying, the morphology of the sEV sample was imaged using a JEX‐1200EX transmission electron microscope (JEOL, Japan) under an acceleration voltage of 100 KV.

### Auditory Brainstem Response Test

Mice were anesthetized through i.p. injection of 10 mg mL^−1^ pentobarbital sodium, dosed at 100 mg kg^−1^ body weight. Subsequently, their ABR thresholds in an open field were assessed using a Tucker‐Davis Technology System III with the BioSigRZ software (TDT, Gainesville, USA). Sound pressure level (SPL) was recorded from 10 to 90 dB in 5 dB increments under six frequencies (4, 8, 12, 16, 24, and 36 kHz). Three electrodes (including ground, reference, and stimulus electrodes) were inserted subcutaneously at the left and right mastoid processes and the vertex cranial, respectively. The hearing threshold was defined as the minimal response level that could consistently observed as a reproducible waveform.

### LC‐MS/MS and Proteomics Analysis

The sEV extracted from ≈50 cochlear explants in each sample were prepared for MS analysis. Subsequently, protein extraction and label‐free LC‐MS/MS analysis were performed by the Beijing Genomic Institute (BGI) (Beijing, China). The data were then analyzed on the Dr. Tom network platform. Briefly, the digested peptides separated by HLPC (UltiMate 3000) (Thermo Fisher Scientific) were detected using a Q‐Exactive HF X (Thermo Fisher Scientific, San Jose, CA), and the data were evaluated using the MaxQuant 1.5.3.30 system integrated with an Andromeda engine performing the search in the Uniport database. A false discovery rate (FDR) ≤ 1% was used for mass filtration at both the spectral level and the protein level to obtain significant identification results. Principal component analysis (PCA) was performed to assess the quality of the data. In addition, MaxQuant was used to extract the intensity of the peak area and thus quantify the protein expression level. The LC‐MS/MS proteomics data have been updated to the ProteomeXchange Consortium through the PRIDE partner repository, with the dataset assigned the identifier PXD052123. To assess the significant variations in protein expression between the Cis‐sEV group and the Ctrl‐sEV group, fold change for different proteins was calculated, and statistical significance was assessed using Welch's t‐test. Furthermore, a fold change (FC) ≥2.0 and Q value (adjusted *p* value^[^ ^90^ ^]^) <0.05 were used as the criteria to determine significant differences. The difference in proteins was presented by volcano plot and difference cluster analysis. In addition, Gene Ontology (GO) analysis using Q values was used for cellular component annotation.

### Small RNA Sequencing and Analysis

The sEVs isolated from ≈80 cochlear explants from each sample were used for small RNA sequencing, which was also performed by BGI (Beijing, China) and analyzed with Dr. Tom's online software. Briefly, total RNA was isolated from sEV samples for library construction. The RNA library was purified and separated from 18–30 nt RNA by PAGE electrophoresis, and then small RNA was reverse transcribed as cDNA and further amplified. After filtering by quality, the small RNA libraries were sequenced on the DNBSEQ platform. The raw data were evaluated and further filtered to obtain clean tags, which were stored in FASTQ format and used for the next analysis. Using Bowtie2 software (with parameters of ‐q ‐L 16 –phred64 ‐p 6), the clean reads were aligned to the GCF_0 00001635.26_GRCm38.p6 reference genome to predict miRNAs and other non‐coding RNA based on their genomic locations. Furthermore, the clean reads were compared against the non‐coding RNA database using AASRA software and against the Rfam database using CMSearch software for annotation of non‐coding RNAs. To avoid having one small RNA compare two different annotations simultaneously and to keep a unique comment, the small RNA was traversed through the comment in order of precedence: MiRbase > Pirnabank > Rfam > other sRNA. The differential expression analysis was calculated based on the MA‐plot (Mean Absolute Deviation plot) and using |log_2_FC| value ≥ 1.0 and Q value ≤ 0.05 as the threshold. The R software with the heatmap function was used for cluster analysis. The small RNA sequencing data presented in this research have been submitted to NCBI's Gene Expression Omnibus and can be accessed via GEO Series accession number GSE266947.

MiRNA target prediction was a necessary process for miRNA function analysis. The RNAhybrid (with parameters of ‐b 100 ‐c ‐f 2,8 ‐m 100 000 ‐v 3 ‐u 3 ‐e ‐20 ‐p 1 ‐s 3utr‐human), miRanda (with parameters of ‐en ‐20 ‐strict), and Target Scan (default parameters) databases were applied to predict target genes of miRNAs in this study. In addition, GO and KEGG pathway analyses were conducted for predicted miRNA targets to analyze their functional annotation and pathway enrichment. After setting the miRNA target minimum score to 3 and the target maximum numbers to 30, the key driver gene analysis (KDA) was performed based on the STRING11 database in the Dr. Tom online system using the default parameters.

### Statistical Analysis

Data were analyzed using the GraphPad Prism 8 statistical software and presented as the mean ± standard deviation (SD) in this study. For statistical analyses between two groups, two‐tailed and unpaired Student's *t*‐tests were conducted. When a *p*‐value < 0.05 was identified as statistically significant. All sEV proteomic and small RNA sequencing analysis was based on Dr. Tom's online platform.

## Conflict of Interest

The authors declare no conflicts of interest.

## Author Contributions

J.A. and S.Z. contributed equally to this work. R.C., S.Z., T.W., and J.A. conceptualized the study and methodology. J.A., M.D., P.J., J.H., H.X., Y.L., X.T., W.T., J.H., Q.M., Y.W. and Z.Y. validated the experiments. J.A. performed the formal analysis, investigation, data curation, and visualization. J.A., S.Z., and T.W. wrote the original draft and performed the review and editing. R.C., S.Z., and T.W. acquired funding. All authors read and approved the final manuscript.

## Supporting information



Supporting Information

## Data Availability

The data that support the findings of this study are available from the corresponding author upon reasonable request.
