# Supplementary material for: Small Extracellular Vesicles Orchestrate Cisplatin‐Induced Ototoxicity: Potential Biomarker and Targets Discovery
Source: Adv Sci (Weinh). 2025 May 24;12(30):e02627. doi: 10.1002/advs.202502627 (PMC12376537; doi:10.1002/advs.202502627)
Supplement: Supplementary file 1 — Supporting Information [file ADVS-12-e02627-s001.docx]

Supplemental information

**Small Extracellular Vesicles Orchestrate Cisplatin-Induced Ototoxicity: Potential Biomarker and Targets Discovery**

Jingru Ai, Shasha Zhang^*^, Mingchen Dai, Pei Jiang, Jingyuan Huang, Hairong Xiao, Yanqin Lin, Xujun Tang, Wei Tong, Jun He, Qiuyue Mao, Yintao Wang, Zixuan Ye, Tian Wang^*^, Renjie Chai^*^


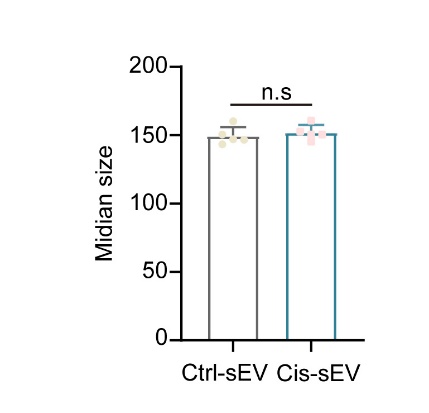


**Figure S1.** Statistical analysis of the median size of the Ctrl-sEV and Cis-sEV. The data are presented as the mean ± SD. The “n.s” represent the no signification. Ctrl-sEV group: n=5 samples; Cis-sEV group: n=5 samples.


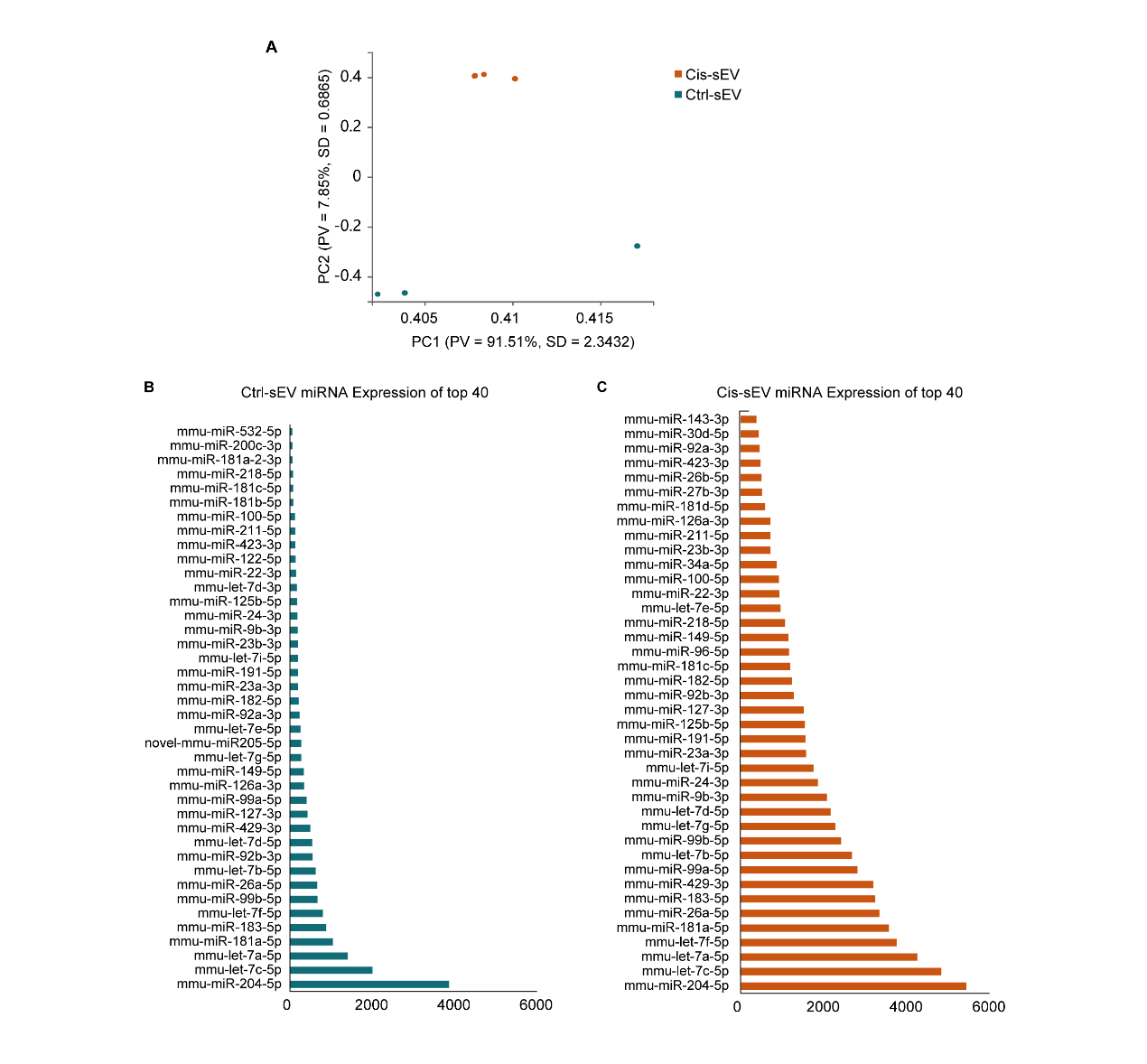


**Figure S2.** (A) Principal component analysis for miRNA-seq in Cis-sEV and Ctrl-sEV. (B, C) Top 40 highest-expressed miRNAs in the Ctrl-sEV and Cis-sEV groups, respectively.


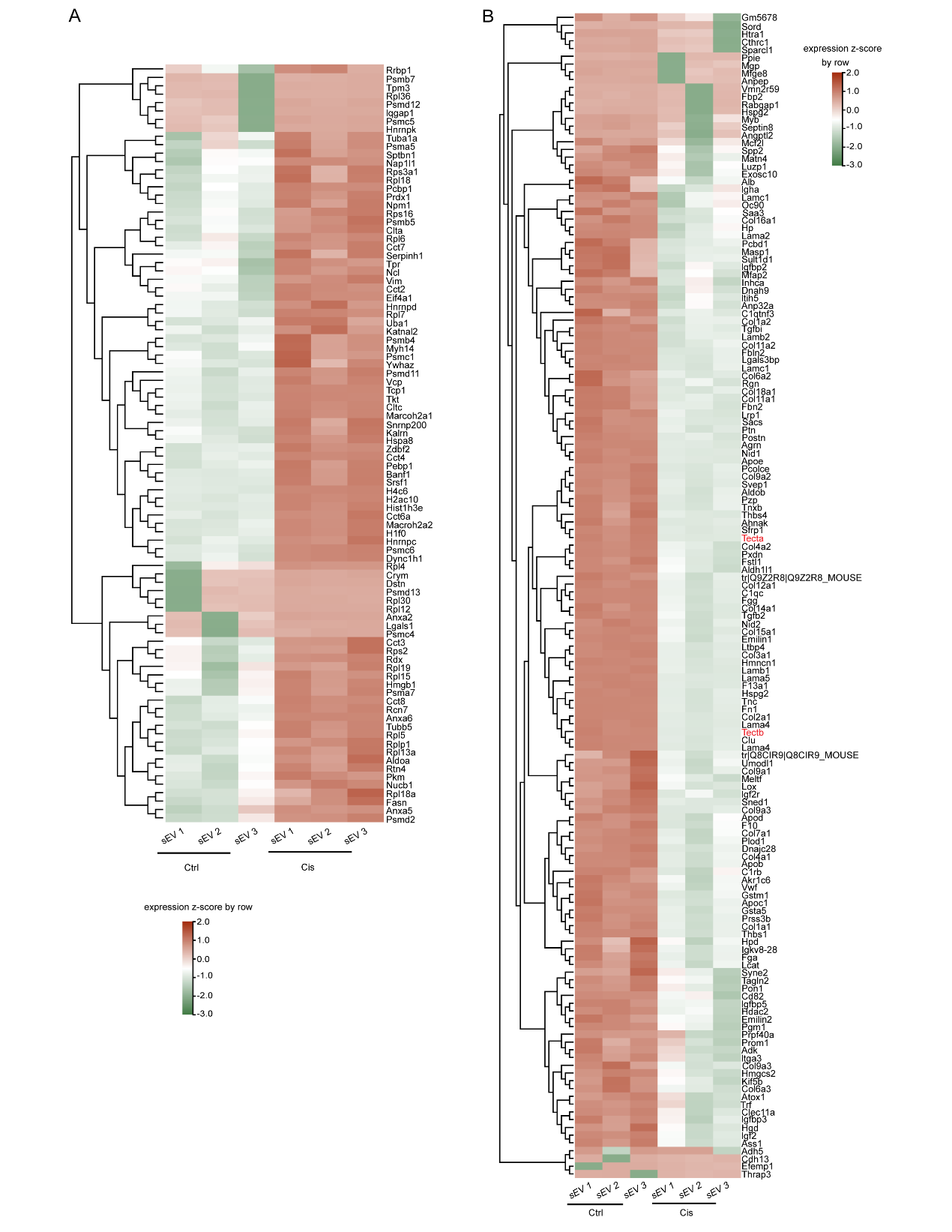


**Figure S****3.** (A, B) Heat map analysis of the 90 upregulated proteins and 150 downregulated proteins in the Cis-sEV group compared to the Ctrl-sEV group. The color bar represents the relative expression level.


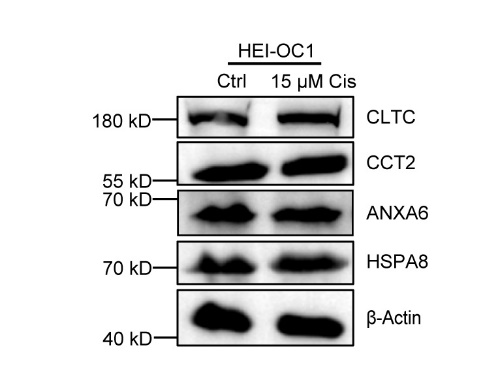


**Figure S4.** Verification of CLTC, CCT2, ANXA6, and HSPA8 was performed in the HEI-OC1 damage model. β-Actin served as the reference protein.

**Table S1.** The novel miRNAs detected in our research with differential abundance expression in Cis-sEV.

| miRNAs | Log2 (Cis-sEV / Ctrl-sEV) | | Sequence (5’-3’) |
| --- | --- | --- | --- |
| novel-mmu-miR121-3p | 11.4419067 | UAACGUGUGAGUGUGUGUUUU | |
| novel-mmu-miR146-5p | 10.55458885 | UUGCUGGGGUGUGCAUGGUGU | |
| novel-mmu-miR208-3p | 6.644065261 | UCCCUCAGACCCUAACUUU | |
| novel-mmu-miR220-5p | 11.31669294 | UGCUAUGAUGAAGGCUAUGUUGGUA | |
| novel-mmu-miR389-3p | 7.957365909 | GCCGAAAGCAUGGGAACAGCC | |
| novel-mmu-miR474-5p | 11.08812569 | CCUGUCUGUGCCUGCUGUAC | |
| novel-mmu-miR68-3p | 11.74188827 | UCCCUGAGACCCUUUAAC | |

**Table S2.** The mRNA RT-qPCR primer sequences were used in this research.

| Gene | Forward sequence (5’-3’) | Reverse sequence (5’-3’) |
| --- | --- | --- |
| *Bax* | TGAAGACAGGGGCCTTTTTG | AATTCGCCGGAGACACTCG |
| *Caspase 3* | CTGACTGGAAAGCCGAAACTC | CGACCCGTCCTTTGAATTTCT |
| *Caspase8* | TGCTTGGACTACATCCCACAC | TGCAGTCTAGGAAGTTGACCA |
| *Caspase9* | TCCTGGTACATCGAGACCTTG | TCCTGGTACATCGAGACCTTG |
| *Tom20* | GCCCTCTTCATCGGGTACTG | ACCAAGCTGTATCTCTTCAAGGA |
| *Bcas3* | CTGACCAGCCAAGACTCTTACA | CTTCGTGATGGTTCCCAGTG |
| *Crb2* | GATCCTAACAGCTTCCGTTGC | GCACTCGTAGTGATCTGCCA |
| *Klhl9* | AAGTCTGGAACTACGCGGTTT | AGCGTCACGTCACAAAGTAGG |
| *Mpp5* | TTTGGGCACCAGAATGATGC | AACAATTCCTTCTTCCGTGTCAA |
| *Ret* | GCATGTCAGACCCGAACTGG | CGCTGAGGGTGAAACCATCC |
| *Usp11* | AACAACATACCGGACGAGGAT | CCTTCATGCCTAGAGGGTTCC |
| *Wwtr1* | CATGGCGGAAAAAGATCCTCC | GTCGGTCACGTCATAGGACTG |

**Table S3.** The primer sequences used for miRNA 1st Strand cDNA synthesis in this research.

| MiRNAs | Stem-loop primer sequence (5’-3’) |
| --- | --- |
| mmu-miR-124-3p | GTCGTATCCAGTGCAGGGTCCGAGGTATTCGCACTGGATACGACGGCATT |
| mmu-miR-140-5p | GTCGTATCCAGTGCAGGGTCCGAGGTATTCGCACTGGATACGACCTACCA |
| mmu-miR-15b-5p | GTCGTATCCAGTGCAGGGTCCGAGGTATTCGCACTGGATACGACTGTAAA |
| mmu-miR-17-5p | GTCGTATCCAGTGCAGGGTCCGAGGTATTCGCACTGGATACGACCTACCT |
| mmu-miR-25-3p | GTCGTATCCAGTGCAGGGTCCGAGGTATTCGCACTGGATACGACTCAGAC |
| mmu-miR-339-5p | GTCGTATCCAGTGCAGGGTCCGAGGTATTCGCACTGGATACGACCGTGAG |
| mmu-miR-34a-5p | GTCGTATCCAGTGCAGGGTCCGAGGTATTCGCACTGGATACGACACAACC |
| mmu-miR-370-3p | GTCGTATCCAGTGCAGGGTCCGAGGTATTCGCACTGGATACGACACCAGG |
| U6 | AACGCTTCACGAATTTGCGT |

**Table S4.** The primer sequences used RT-qPCR for miRNA in this research.

| MiRNAs | Forward sequence (5’-3’) ^a)^ |
| --- | --- |
| mmu-miR-124-3p | GCGTAAGGCACGCGGTG |
| mmu-miR-140-5p | CGCGCAGTGGTTTTACCCTA |
| mmu-miR-15b-5p | CGCGTAGCAGCACATCATGG |
| mmu-miR-17-5p | GCGCAAAGTGCTTACAGTGC |
| mmu-miR-25-3p | GCGCATTGCACTTGTCTCG |
| mmu-miR-339-5p | CGTCCCTGTCCTCCAGGAG |
| mmu-miR-34a-5p | CGCGTGGCAGTGTCTTAGCT |
| mmu-miR-370-3p | GGCCTGCTGGGGTGGAA |

^a)^ The Reverse primers and U6 primers are provided in the miRNA Unimodal SYBR qPCR Master Mix Kit (Vazyme, MQ102).
